# Biodiversity management of organic farming enhances agricultural sustainability

**DOI:** 10.1038/srep23816

**Published:** 2016-04-01

**Authors:** Haitao Liu, Jie Meng, Wenjing Bo, Da Cheng, Yong Li, Liyue Guo, Caihong Li, Yanhai Zheng, Meizhen Liu, Tangyuan Ning, Guanglei Wu, Xiaofan Yu, Sufei Feng, Tana Wuyun, Jing Li, Lijun Li, Yan Zeng, Shi V. Liu, Gaoming Jiang

**Affiliations:** 1 State Key Laboratory of Vegetation and Environmental Change, Institute of Botany, The Chinese Academy of Sciences, Nanxincun 20, Xiangshan, 100093, Beijing, China; 2 University of the Chinese Academy of Sciences, No. 19, Yuquan Avenue, 100049, Beijing, PR China; 3 Research Center for Eco-Environmental Sciences, The Chinese Academy of Sciences, 18 Shuangqing Road, Haidian District, 100085, Beijing, China; 4 Institute of Geographic Sciences and Natural Resources Research, The Chinese Academy of Sciences, 11A, Datun Road, Chaoyang District, 100101, Beijing, China; 5 State Key Laboratory of Crop Biology, Shandong Agricultural University, No. 61, Daizong Avenue, 271018, Tai’an, China; 6 Hongyi Organic Farm, 273300, Pingyi, China; 7 Eagle Institute of Molecular Medicine, Apex, NC, USA

## Abstract

Organic farming (OF) has been believed to be capable of curtailing some hazardous effects associated with chemical farming (CF). However, debates also exist on whether OF can feed a world with increasing human population. We hypothesized that some improvements on OF may produce adequate crops and reduce environmental pollutions from CF. This paper makes comparative analysis of crop yield, soil organic matter and economic benefits within the practice on Biodiversity Management of Organic Farming (BMOF) at Hongyi Organic Farm (HOF) over eight years and between BMOF and CF. Linking crop production with livestock to maximal uses of by-products from each production and avoid xenobiotic chemicals, we have achieved beneficial improvement in soil properties, effective pest and weed control, and increased crop yields. After eight years experiment, we have obtained a gradual but stable increase in crop yields with a 9.6-fold increase of net income. The net income of HOF was 258,827 dollars and 24,423 dollars in 2014 and 2007 respectively. Thus, BMOF can not only feed more population, but also increase adaptive capacity of agriculture ecosystems and gain much higher economic benefits.

It has been widely circulated that coming decades will see a large increase of human population from seven billion now to nine billion in 2050^1^, with a potential of doubling the food demand^2^. To meet this anticipated challenge, various options have been proposed and tested^1^,^2^. With wide application of chemical fertilizers, pesticides and herbicides, large-scale utilization of water resources, and implementation of genetic engineering, the global per capita food production has been increased significantly^2^,^3^. However, these efforts have also resulted in some negative impacts on environment and biodiversity and thus present potential threat to the global ecosystem^3^,^4^,^5^. Soil contamination^6^ and acidification^7^, groundwater pollution^8^, and elevated land emission of greenhouse gases^9^ are commonly associated with chemical farming (CF) areas. The aggressive use of synthetic pesticides and herbicides has not achieved the ideal goal of long-lasting control of harmful insects and weeds but resulted in some decrease of the natural enemies of the harmful insects^10^,^11^ and even some increases of herbicide-resistant super weeds^12^,^13^. Moreover, the loss of biodiversity can reduce crop production in return. A meta-analysis of published data showed that intermediate levels of species loss (21–40%) reduced plant production by 5–10% while higher levels of extinction (41–60%) had adverse effects rivalling those of ozone, acidification, elevated CO_2_ and nutrient pollution^14^.

What should we do to reduce negative environmental impacts of CF and increase food production in an ecologically-beneficial way? Some have conducted innovative work to produce more grain with lower environmental costs^7^,^15^,^16^,^17^. There may be even more economic and ecological benefits to gain if crop production is effectively linked with livestock production in the same agricultural system. In this regard, we might believe that organic farming (OF) traditionally practiced in small scale by farmers like those in China can be improved and re-organized. By doing so, we may maximally utilize by-products of crop production and livestock production to enhance crop yield and reduce environmental contaminations^18^.

We hypothesized that, if crop production can be effectively linked with livestock production in the same agricultural system, not only the yields of crops might be increased without chemical pollutions but also some economic and ecological benefits could be gained. Thus, we established in 2006 an organic farm, Hongyi Organic Farm (HOF), to test the above hypothesis. HOF has a total of 8.7 ha of lands located in Shandong Province of East China. Biodiversity Management of Organic Farming (BMOF) practiced in HOF (Fig. 1) included feeding cattle with crop residues, and using manure produced from earthworm-digested composting cattle dung as fertilizer. Weeds were controlled by mulching farming land with chopped straws and or labor. We employed sola-powered pest-trapping lights and natural enemies for capturing flying insects and pests during night times. Chicken farming was carried out in forest and organic apple orchard. We did not use any chemical fertilizer, pesticide, or herbicide. We also avoid planting any genetically modified crops (GMCs). Reported here are initial results of practicing BMOF in HOF for eight years.

## Results

### Increasing crop yields with BMOF

**Figure 2:**
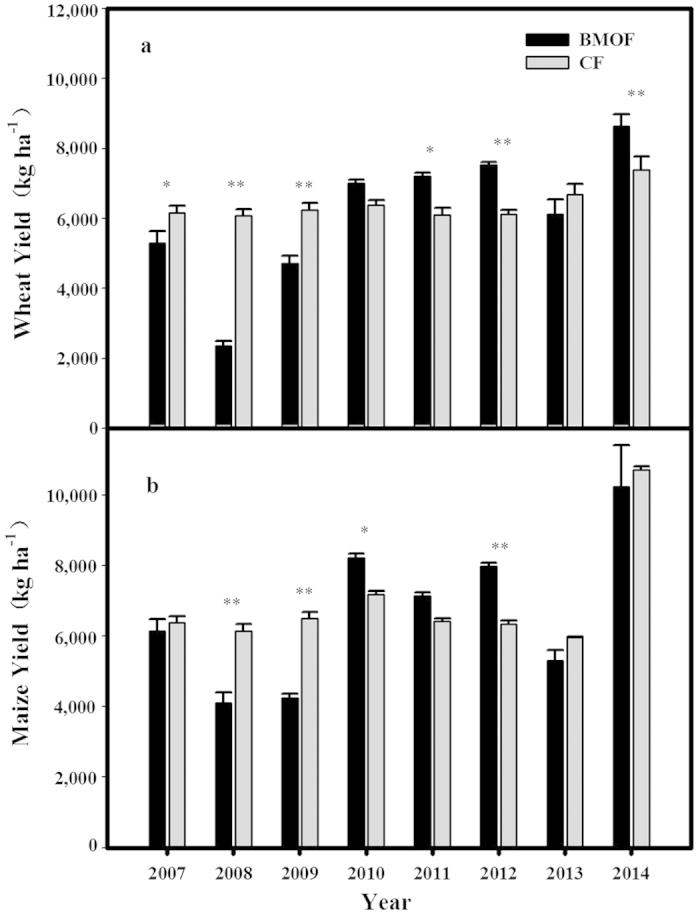
Comparison of winter wheat (**a**) and summer maize (**b**) yields from 2007 to 2014 between Biodiversity Management of Organic Farming (BMOF) and chemical farming (CF). The different footnote symbols represent significant difference (DUNCAN test, **extremely striking contrast (*P* < 0.01); *significant difference (*P* < 0.05)). In the organic transition period (2007–2009), crops yield of BMOF was lower than CF. However, BMOF yields got a gradual but stable increase both in winter wheat and summer maize after transition period and better performed in the later years than CF.

The BMOF has resulted in a consistent increase of crop yields for winter wheat (Fig. 2a) and summer maize (Fig. 2b) up to 65% per ha over eight years. More importantly, even higher crop yields were obtained at the end of eight years’ BMOF in HOF than the adjacent farm lands with CF (Fig. 2). At the 8^th^ year of the experiment, the winter wheat yield and summer maize yield were 8,634 kg ha^−1^ and 10,235 kg ha^−1^, respectively, in the BMOF lands as compared with about 7,394 kg ha^−1^ and 10,715 kg ha^−1^ in the CF lands. The slightly lower yields (about 4.3% less) in the BMOF lands than the adjacent CF lands in the eight years average may not be a substantially difference considering the overall higher yields (8.7% more) in the BMOF lands than the adjacent CF lands for the last five years of the experiment.

### Improved soil quality with earthworm-digested compost

**Figure 3:**
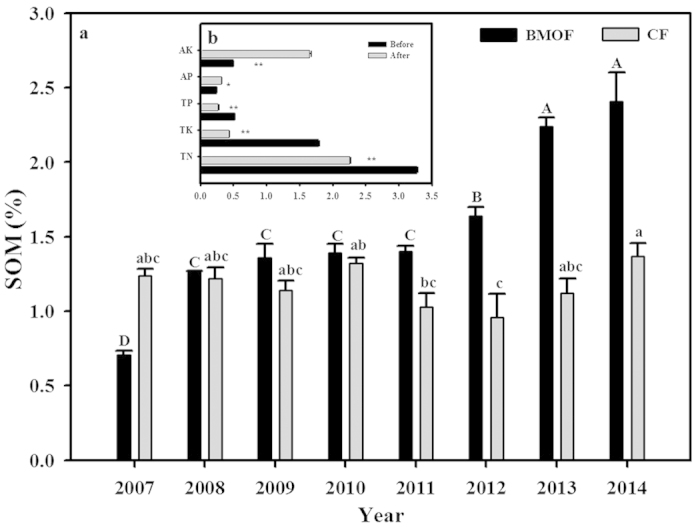
Changes in soil properties as reflected in top soil organic matter (SOM, 0–20 cm) (**a**) and mineral elements contents before and after earthworm digested compost (**b**). AK, content of total available potassium (g kg^−1^); AP, rapidly available phosphorus (g kg^−1^); TP, content of total phosphorus (%); TK, content of total potassium (%); TN, total nitrogen (%). The different footnote symbols represent significant difference (DUNCAN test, **extremely striking contrast (*P* < 0.01); *significant difference (*P* < 0.05)). Same letter(s) means no significant difference (*P* < 0.05), lower-case for chemical farming (CF) and upper-case for Biodiversity Management of Organic Farming (BMOF).

As shown in Fig. 3a, the sum of soil organic matters (SOM) in 0–20 cm depth layer increased from 0.7% to 2.4% over the experimental run. In contrast, the SOM of CF lands has no significant (*P* = 0.3050) change during this time period. The contribution of earthworm-digested compost to this conversion of previously barred lands into productive lands was apparent. Although the earthworm-digested compost has lower contents of total nitrogen, phosphorus and potassium compared to the original cattle manure, the available phosphorus (*P* = 0.0409) and potassium (*P* = 0.0031) are significantly higher (Fig. 3b).

### Non-toxic pest trapping

**Figure 4:**
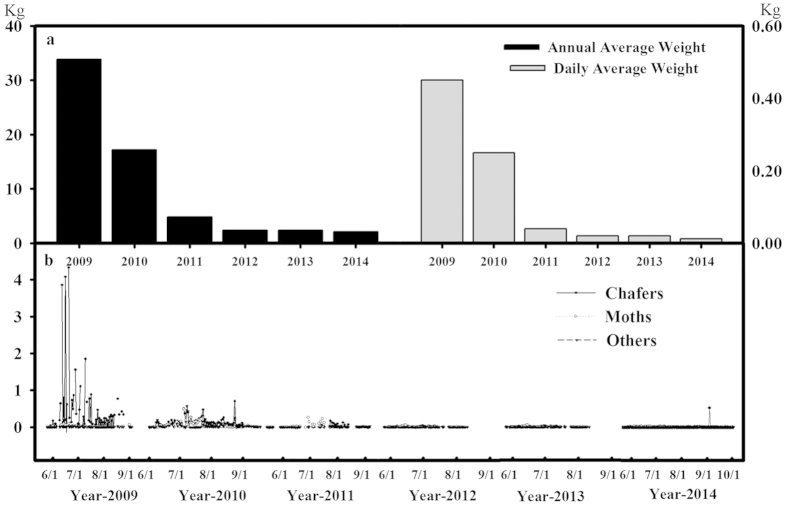
Annual weight reduction (**a**) and dynamic changes (**b**) of fresh weight of chafers and months monitored by the solar pest-trapping lights from 2009 to 2014. The lights were automatically switched on daily from 7 pm to 5 am during the night time from May to September.

Effective pest control has been achieved using pest-trapping lights (Fig. 4). Both daily and annual average fresh weight of trapped pests reduced substantially with each passing year. We noted a considerable decline in average total fresh weight of trapped pests, from 0.45 kg day^−1^ in 2009 to 0.012 kg day^−1^ in 2014. The annual total fresh weight of trapped pests has been reduced by 93.8%. Detailed examinations showed that this decreased catching of pests included reduced catch for both chafers and moths (Fig. 4b), with only 2.4 kg and 2.1 kg trapped per light in 2013 and 2014, respectively, as compared with 38.4 kg in 2009. Thus the annual total weight of trapped pests has been reduced from 33.8 kg to 2.1 kg (Fig. 4a), demonstrating the effectiveness of this pest control approach.

### Overall economic benefits

It is worthy to note that, after eight years of BMOF practice, we have obtained a 9.6-fold increase of net income on a whole farm scale against the beginning year. The net income of HOF was 258,827 dollars and 24,423 dollars in 2014 and 2007 respectively (Table 1). As a matter of fact, while the total inputs in BMOF increased 368% when compared with CF, the total outputs increased by 408%. The per unit area net benefits from BMOF was 5 times higher than that from CF (Supplementary Table S1). However, according to our investigation, just crop planting inputs per ha from the organic farm are almost equal to those of the conventional one (3019 $ ha^−1^ VS. 2806 $ ha^−1^, Supplementary Table S2).

## Discussion

Most scientific literature claimed that OF has an average 19.8–25% lower yield than CF^19^,^20^,^21^. However, this is not the case for all crops and all climate zones^20^,^22^. When using best management practices, the OF yield can match with or improve over CF yield^20^,^21^. Our eight years of BMOF experiment provides an actual example for increasing crop yield with just OF. This is because we not only obtained higher yields in the later years than the earlier years on the same lands with BMOF but also achieved even higher crop yields in the BMOF lands than that reported by the adjacent lands still practicing CF (Fig. 2).

The increased crop yields of BMOF lands might be contributed by improved soil quality as well as by effective weed control and insect/pest control. Through BMOF of applying chopped straws for mulching and earthworm-digested cattle manure as organic fertilizers the SOM in the previously abandoned lands has been greatly increased (from 0.7% to 2.4%, Fig. 3). Earthworms are well known to benefit soil nutrients via accelerating decomposition and mineralization nutrients from organic residues^23^,^24^. The essential minerals to plant growth such as nitrogen, potassium, phosphorus, and calcium are much more soluble and available to plants through earthworm composting^25^. In addition, through earthworm composting, the bioavailability of the essential minerals in the manure also increased significantly^25^.

BMOF practice also helps to avoid the cost of purchasing pesticides, herbicides and animal feeds. The usage of pest-trapping lights not only reduces the cost and labor for the pest management but also add values to husbandry practiced on site. Using pests trapped in a non-toxic way via solar-powered trapping lights for feeding chickens raised in the BMOF lands, a considerable economic benefit was also gained. The on-site livestock husbandry in return industry contributed more to the soil improvement by discharging organic fertilizer back into the same land.

Without using any herbicides while just using chopped straws to mulch exposed land, we effectively controlled weeds during summer maize season. However, weed management still needs more labors. But this shortcoming of BMOF practice may not present any problem but rather provide a solution to China’s agricultural system because a large number of Chinese farmers actually often have to contend for a limited land to work. When OF not only produces enough crops but also bring back some economic values, more famers may enjoy their beneficial agricultural work and thus relieve society some burden for creating other employments for them.

We should point out that the net cost and thus the benefits of organic farming may vary among different countries because the material cost and manpower charge are different in different economic systems. The organic products we sold are 3–5 times higher than conventional ones.

Agricultural biodiversity is a key for a better performance of BMOF. With a management of biodiversity, we found that OF can not only feed more population, but also enhance adaptive capacity of agriculture ecosystems^26^, mitigate greenhouse gas emissions^17^ and reduce environmental pollutions. BMOF is not only an ecologically attractive approach but also an economically beneficial means which should be fully considered for establishing sustainable agricultural system. Considering the increasingly serious chemical pollution from excessive CF^27^ and the resistance to GMCs due to various concerns such as their potential health risks^28^,^29^, we further believe that OF is a practical alternative that can bring long term benefit.

Thus, rather than continuing in pushing global environmental change into a chemical-polluted geological epoch of “Anthropocene”^30^, we should extend more BMOF to build an ecosystem-beneficial, environment-friendly and human-healthy sustainable society. This approach may be a practical route for better utilization of a large population of farmers on a limited size of farming lands. It may also provide a valuable means for realizing the sustainable development goals (SDGs) for the Millennium Development Goals (MDGs) set up by the United Nations (UN) aimed at assuring the stability of Earth’ systems while ending the poverty and hunger of human society^30^.

## Methods

### The study area

Hongyi Organic Farm (HOF) was established in 2006 on a total of 8.7 ha area taken from local farmers who had abandoned the land for years due to its poor crop-growing properties. The farm has 6.7 ha crop land, 0.3 ha apple orchard, 1.3 ha cattle houses and 0.4 ha forest. The farm is physically located in Jiangjiazhuang Village (35°26′21″N, 117°50′11″ E), Shandong Province, East China. This locality has a temperate and monsoonal climate with four clearly distinct seasons. The average annual temperature is 13.2 °C and the mean annual precipitation is 770 mm. The conventional methods for weed and pest control in the village are through spraying herbicide and pesticide. The total crop acreage in the village is 33.3 hm^2^, producing 738.8 tons fresh crop residues (mainly wheat and corn stalks) every year with most of them discarded or directly burned in the field.

### Crop rotation and management

The crop planting system was based on wheat (*Triticumaestivium* L.)-maize (*Zea mays* L.) rotating plantation. The winter wheat variety was Liangxing 99, and the summer maize was Zhengdan 958. Winter wheat was sown in early October and harvested in early June of the next year. Summer maize was sown in June and harvested at the end of September or beginning of October. The usage of wheat seed was 225 kg ha^−1^, and the density of maize was around 55,000 plant ha^−1^. In HOF, we abandoned chemical fertilizers, pesticide, and herbicide, with chemical fertilizers being replaced by earthworm-digested manure (75 t ha^−1^, composed fresh weight). For CF, fertilizers for winter wheat were used at the rate of 225 kg N ha^−1^, 750 kg P_2_O_5 _ha^−1^ and 150 kg K_2_O ha^−1^, respectively. And fertilizers for summer maize in CF was used at the rate of 150 kg N ha^−1^, 600 kg P_2_O_5 _ha^−1^ and 210 kg K_2_O ha^−1^, respectively. Crops were irrigated twice each year respectively in winter and spring season (only in the wheat season) for both BMOF and CF. Crop land was 6.7 ha and 8.7 ha for BMOF and CF, respectively. Winter wheat yield was determined by actually harvesting three randomly chosen 1 m^2^ quadrants. For corn, three rows of 15 continuous plants were harvested. Crops were harvested with the seed water content being <14%.

### Pest control and weeds management

To replace the chemical pesticides, solar pest-trapping lights were introduced. Since 2009, a long-term pest monitor station was built in HOF, with insects being trapped and weighed daily during the growth seasons. The trapping lights utilized a 365 ± 50 nm wavelength spectrum and purple color light to attract and trap adult pests including moths (*Ostrinia nubilalis* and *EuxoasegetumSchiffer-muller*) and chafers (*Holotrichia parallela Motschulsky* and *Holotrichia diomphalia Bates*). Once trapped, the pests were electrocuted by the high voltage electricity generated by the device. The lights were automatically switched on daily from 7 pm to 5 am during the night time from May to September, in accordance with the growing period of crops. Then the moths and chafers were used for feeding chickens. Straw mulching was used to control weed in the organic farm. When crop harvested, straw were chopping into small pieces by machine and returned into the field. Compared with BMOF, CF used pesticides and herbicides with 2.1 kg ha^−1^ in total.

### Crop residues processing and residues-based livestock production

Corn residues were machine processed into silages at the harvest stage. The crushed and kneaded crop residues were pressed and framed in a large silage pool (500 m^3^). Then the pool was covered with plastic and stored for natural fermentation. The feeding cattle experiment was conducted in the farm for 7 months from November of 2007 to June of 2008. The livestock average starting weight was at about 220 kg head^−1^. The average fine fodder input is 2.5 kg (including maize meal, cottonseed meal, wheat bran, baking soda, cod liver oil etc.) per cattle per day. Silage was used as roughage. The consumption of silage varied along with the cattle’s growth stage. Data was collected daily for final analysis during the experimental run.

### Earthworm-digested compost

Cattle dung was shaped into 50 cuboids with width 1.5 m and height 0.3 m with an initial earthworm population density of 0.5 kg m^−2^. 11 traps had been done according to above method^31^. The cattle dung was watered regularly to keep a proper humidity. The earthworm and cattle manure were separated after 3 months. The manure sample was collected for final chemical analysis. The digested manure was used as organic fertilizer.

### Soil and earthworm-digested compost sampling and analysis

Soils were sampled with three replications in October every year (after maize harvest) for both BMOF and CF, where five individual cores were taken randomly in 0–20 cm soil depth layer and then mixed into one replication. The litter, loose stoned or plant shoots were removed before sampling. The soil samples were dried, air sieved to <2 mm, and then grounded for the analysis of soil organic matter (SOM). The organic matter content was determined by the dichromate oxidation method^32^. The earthworm-digested compost sample was collected in different depths and thereafter bulked to one composite compost sample after composting completed. Compost samples were passed through a 10 mm sieve to remove any oversized material. The manure sample was dried in air and then grounded for the analysis of total N (TN), total P (TP) and total K (TK) and cattle manure was measured before and after the earthworm digestion. TN was determined by semi-micro Kjeldahl + NO^3−^-N, according to Laoset^33^ and Tognetti^34^. Total P and K were extracted by the nitric-perchloric digestion (1:1) and analyzed according to EPA Method 3050^35^. Available-K was determined by flame photometry after extraction with 1 M ammonium acetate at pH 7^36^; available-P was extracted with 0.5 M NaHCO_3_ (1:10, w/v) for 30 min and measured colorimetrically^37^.

### Farm scale economic assessment

Total economic benefits of BMOF included crop yield benefits, cattle breeding benefits, apple orchard benefits, poultry benefits (chicken and goose) and cash forest benefits while only crop yield benefits for CF. Net benefits were calculated on the whole farm scale from 8.7 ha land in total and per unit area for both BMOF and CF. Detailed data of every category was showed in Supplementary Tables. The local prices were different in years such as labor, young cattle, young chicken, young goose and beef.

### Data analyses

Crop yields data for analysis were up-scaled to one hectare based on the data collected from our farm and adjacent farm lands. Crop yield and soil data were analyzed using one-way analysis of variance (ANOVA) of SAS statistics for windows. Differences in the treatments were compared using DUNCAN test. The significance level was set at *P* < 0.05. Figures were generated with using Sigma Plot 10.0 (Aspire Software Intl. Ashburn, VA, USA).

### Ethics Statement

The field site was owned by the Agricultural Ecosystem Research Station of Shandong Agricultural University. We obtained the permission to conduct the study from the Station. This study field did not involve endangered or protected species and it is located in a pure agricultural area. This study was carried out in strict accordance with recommendations made in the Guide for the Care and Use of Laboratory Animals of the Chinese Academy of Science. The protocol of feed animals was approved by the Chinese Academy of Sciences (Permit Number: KZCX2-YW-Q1-13). There was not any surgery performed on animals during the experiment. Following the experiment, cattles were lagally sold to a local licensed business man.

## Additional Information

**How to cite this article**: Liu, H. *et al*. Biodiversity management of organic farming enhances agricultural sustainability. *Sci. Rep.* **6**, 23816; doi: 10.1038/srep23816 (2016).

## Supplementary Material

Supplementary Information

## Figures and Tables

**Figure 1:**
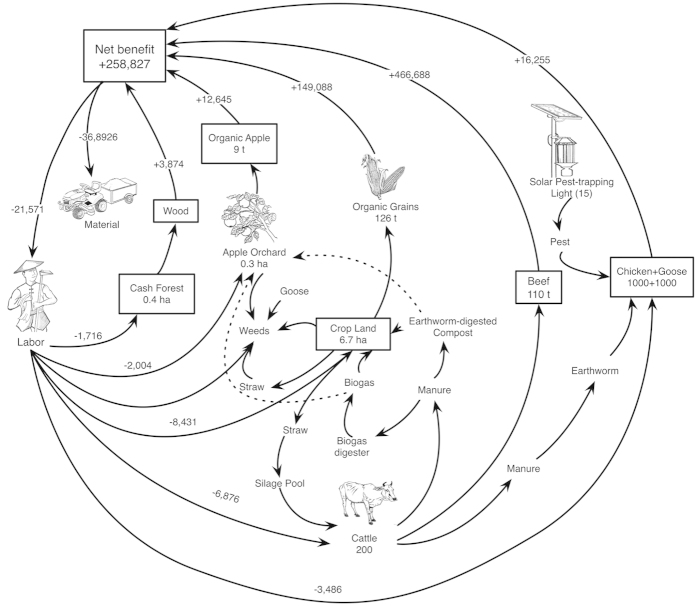
A schematic diagram of Biodiversity Management of Organic Farming (BMOF) practice in Hongyi organic farm (HOF). The ‘+’ means money outputs, ‘−’ means money inputs. Money unit is US dollar. All the data showed in this diagram is collected in the eighth year of BMOF on the whole farm scale. This system consists of crops, apple orchard, cattle, forest, chicken and goose. The land areas of crop, orchard, cattle breeding and cash forest are 6.7 ha, 0.3 ha, 1.3 ha and 0.4 ha, respectively. Chicken and goose are free-range fed under forest and apple. Total inputs are separated into labor and materials cost. Materials include machine depreciation, fertilizer, irrigation, seeds, electricity, etc. BMOF in HOF has five main material circles, including: 1) feeding cattle with crop residues; 2) using manure produced from earthworm-digested composting cattle dung as fertilizer; 3) weed management by mulching farming land with chopped wheat straws and or labor; 4) pest management through employing solar light traps and natural enemies; 5) chicken farming in forest and organic apple orchard. All the drawing in this figure was drawn by Da Cheng.

**Table 1:** Total economic benefit, crop yield benefits, cattle benefits, orchard benefits and poultry benefits (chicken and goose) during 2007 to 2014 in Biodiversity Management of Organic Farming (BMOF) practices.

Years	Crops§($)			Orchard§($)			Cattle§($)			Chicken+goose§ ($)			Cash forest§			Total net benefit
	Inputs*	Outputs**	Net outputs	Inputs*	Outputs**	Net outputs	Inputs*	Outputs	Net outputs	Inputs*	Outputs	Net outputs	Inputs*	Outputs	Net outputs	
2007	32,421	41,423	9,002	6,771	6,232	−539	39,929	54,533	14,604	9,683	11,981	2,298	942	0	−942	24,423
2008	25,984	24,009	−1,975	6,771	6,415	−356	54,231	76,485	22,254	13,751	24,469	10,718	0	0	0	30,641
2009	30,481	31,878	1,397	7,104	6,901	−203	91,292	162,328	71,037	9,683	9,038	−443	0	0	0	71,787
2010	38,794	120,294	81,500	7,104	13,777	6,673	108,580	216,098	107,519	8,353	8,683	330	0	0	0	196,021
2011	37,567	114,254	76,687	7,104	13,840	6,736	176,709	252,115	75,406	11,330	14,525	3,195	0	0	0	162,024
2012	40,504	123,021	82,517	7,438	11,261	3,823	190,267	323,385	133,796	11,330	15,544	4,214	0	0	0	224,348
2013	35,086	91,427	56,341	7,438	11,953	4,515	292,582	420,019	127,437	12,815	16,096	3,281	0	0	0	191,575
2014	44,993	149,088	104,095	7,438	12,645	5,207	324,477	466,688	142,211	12,815	16,255	3,440	774	4648	3,874	258,827

Unit: US$.

^§^The details of every category were showed in supplementary tables. The land areas of crop, orchard, cattle breeding and cash forest are 6.7ha, 0.3ha, 1.3ha and 0.4ha, respectively.

^*^The labor price was different in different years as showed in Supplementary Table S5.

^**^We sold the grains and apple as ordinary food during 2007–2009 because the conversion period to organic is generally three years. The price of ordinary food is showed in Supplementary Table S2 and Supplementary Table S3, respectively for grain and apple.
