# Supplementary material for: Biodiversity management of organic farming enhances agricultural sustainability
Source: Sci Rep. 2016 Apr 1;6:23816. doi: 10.1038/srep23816 (PMC4817119; doi:10.1038/srep23816)
Supplement: Supplementary Information [file srep23816-s1.pdf]

Biodiversity management of organic farming enhances agricultural sustainability

Haitao Liu[1,2], Jie Meng[1], Wenjing Bo[3], Da Cheng[1], Yong Li[4], Liyue Guo[1], Caihong Li[1], Yanhai Zheng[1], Meizhen Liu[1], Tangyuan Ning[5], Guanglei Wu[1], Xiaofan Yu[1], Sufei Feng[5], Tana Wuyun[5], Jing Li[1], Lijun Li[1], Yan Zeng[6], Shi V. Liu[7], Gaoming Jiang[1,5*].

[1]State Key Laboratory of Vegetation and Environmental Change, Institute of Botany, The Chinese Academy of Sciences, Nanxincun 20, Xiangshan, 100093, Beijing, China.

[2]University of the Chinese Academy of Sciences, No.19, Yuquan Avenue, 100049, Beijing, PR China

[3]Research Center for Eco-Environmental Sciences, The Chinese Academy of Sciences, 18 Shuangqing Road, Haidian District, 100085, Beijing, China.

[4]Institute of Geographic Sciences and Natural Resources Research, The Chinese Academy of Sciences, 11A, Datun Road, Chaoyang District, 100101, Beijing, China.

[5]State Key Laboratory of Crop Biology, Shandong Agricultural University, No. 61, Daizong Avenue, 271018, Tai'an, China.

[6]Hongyi Organic Farm, 273300, Pingyi, China.

[7]Eagle Institute of Molecular Medicine, Apex, NC, USA.

*Correspondence to: E-mail: jgm@ibcas.ac.cn(G.M. Jiang)

Supplementary Materials list:

Supplementary Tables S1-S9

Tables S1 Comparison of net benefits per unit area between Biodiversity Management of Organic Farming (BMOF) and chemical farming (CF).

Content	BMOF	CF
Crop land (ha)	6.7	8.7
Apple orchard (ha)	0.3	0
Cattle breeding land (ha)	1.3	0
Cash forest (ha)	0.4	0
Total land (ha)	8.7	8.7
8-year average labor inputs ($ ha^{-1})	1,763	974
8-year average total inputs ($ ha^{-1})	24,596	5,251
8-year average total outputs ($ ha^{-1})	36,489	7,186
8-year average total net benefits ($ ha^{-1})	11,893	1,935
Net benefits ratio of BMOF to CF (BMOF/ CF)	6.1	

Tables S2 Detailed list of crop inputs and outputs for Biodiversity Management of Organic Farming (BMOF) and chemical farming (CF).

Details of Crop Inputs				
**Content**	Details	Price Schedules	BMOF Amount ($ ha^{-1})	CF Amount ($ ha^{-1})
**Machine**	Transportation fare for organic fertilizer	3.23 $ CAR^{-1} *22.5 CAR ha^{-1}	72.6	0.0
	Rotary tillage fare before winter wheat seeding	Mechanical rotary tillage,121.05 $ ha^{-1}	121.1	121.1
	Fare for soil preparation and seeding	96.84 $ ha^{-1}	96.8	96.8
	Combine harvester for winter wheat	193.68 $ ha^{-1}	193.7	193.7
	Combine harvester for corn	193.68 $ ha^{-1}	193.7	193.7
	Fare for corn thresher	12.11 $ ha^{-1}	12.1	12.1
	Solar energy light-traps depreciation costs	322.8 $ $light^{-1}$, 1 light ha^{-1}, 5 years for 1 light	64.6	0.0
**Sum**			**755**	**617**
**Irrigation**	Requirements for irrigation to government	217.89 $ ha^{-1}*2	**436**	**436**
**Labor**	Composting	2 labor ha^{-1} , 9.68 $ $labor^{-1}$	19.4	19.4
	Loading and tedding Organic fertilizer	15 labor ha^{-1}	145.3	0.0
	Irrigation	9 labor ha^{-1}	87.1	87.1
	Spray insecticide for winter wheat	4.5 labor ha^{-1}	0.0	43.6
	Air basks in wheat	7.5 labor ha^{-1}	72.6	72.6
	Corn artificial seeding	18 labor ha^{-1}	174.3	174.3
	Spray herbicide for corn	7.5 labor ha^{-1}	0.0	72.6
	Spray insecticide for corn	6 labor ha^{-1}	0.0	58.1
	Drainage for corn	4.5 labor ha^{-1}	43.6	43.6
	Manual weeding	15 labor ha^{-1}	145.2	0.0
	Corn harvest	18 labor ha^{-1}	174.4	174.4
	Cleaning up cornstalk	21 labor ha^{-1}	203.3	203.3
	Peeling of corn	15 labor ha^{-1}	145.2	145.2
	Corn threshing	2 labor ha^{-1}	19.4	14.5
	Air basks in corn	3 labor ha^{-1}	29.1	29.1
**Sum**		130 labor ha^{-1}	**1,259**	**1,138**
**Fertilizer**	Cost for organic fertilizers	75cube ha^{-1},4.84$ m^{-3}	363.2	0.0
	Cost for chemical fertilizers	14.53 $ for winter wheat,8.07 $ for corn	0.0	338.9
**Sum**			**363**	**339**
**Pesticide**	Pesticides for wheat seed dressing	30 bag ha^{-1},0.4 $ bag^{-1}	0.0	12.1
	Wheat aphids pesticide	15 bottle ha^{-1}, 1.6 $ $bottle^{-1}$	0.0	24.2
	Herbicides for corn	24.21 $ ha^{-1}	0.0	24.2

	Pesticides for corn borer	30 bag ha^{-1}, 0.32 \$ bag^{-1}	0.0	9.7
**Sum**			**0**	**70**
**Seeds**	Cost for winter wheat seed	0.48 \$ kg^{-1},225 kg ha^{-1}	109.0	109.0
	Cost for corn seed	3.23 \$ kg^{-1}, 30 kg ha^{-1}	96.8	96.8
**Sum**			**206**	**206**
**Total**			**3,019**	**2,806**
**Details of Flour Production Inputs**				
	Details	BMOF (\$ kg^{-1})	CF (\$ kg^{-1})	Extraction Rate (%)
**Flour Production**	Grinding wheat flour	0.023	0.023	75
	Grinding corn flour	0.045	0.045	60
	Flour bag	0.161	0.006	
**Details of Crop Outputs**				
	Details	BMOF (\$ kg^{-1})	CF (\$ kg^{-1})	
**Products Price**	Wheat flour price	1.62	0.52	
	Corn flour price	1.62	0.82	
	Grain bran price	0.29	0.29	

TableS3. Detailed list of apple orchard inputs and outputs for Biodiversity Management of Organic Farming (BMOF).

Details of apple orchard Inputs			
**Content**	Details	Price Schedules	Amount ($ ha^{-1})
**Fertilizer**	Costs for manure composts	217.5 cube ha^{-1}, 4.84 $ m^{-3}	1,053
**Sum**			**1,053**
**Transportation fare**	Transportation fare for manure compost	1.61 $ car^{-1}, 150 car ha^{-1}	242.1
	Transportation fare for biogas slurry	12.9 $ car^{-1}, 9 car ha^{-1}	116.2
**Sum**			**358**
**Labor**	Scrape old bark	7.5 labor ha^{-1}, 9.68 $ labor^{-1}	72.6
	Pruning	30 labor ha^{-1}	290.4
	Clean up the branches on the ground	15 labor ha^{-1}	145.2
	Bagging	75 labor ha^{-1}	726.0
	Blossom and fruit thinning	60 labor ha^{-1}	580.8
	Irrigation	15 labor ha^{-1}	145.2
	Bag removing	30 labor ha^{-1}	290.4
	Harvest	30 labor ha^{-1}	290.4
	Pest control	90 labor ha^{-1}	871.2
	Manure compost application	165 labor ha^{-1}	1,597.2
	Weeds management	30 labor ha^{-1}	290.4
	Planting *Duchesneaindica*	7.5 labor ha^{-1}	72.6
**Sum**		**690 labor ha^{-1}**	**6,680**
**Irrigation**	Requirements for irrigation to government	217.89 $ha^{-1}*4	**872**
**Pest control**	lime sulphur		113.5
	burgundy mixture	115 $ per time, 3 times	345.0
	Biogas slurry	36.3 $ per time, 3 times	108.9
	sticky card		145.3
	Stem residue trap		169.5
	Solar energy light-traps depreciation costs (5 years)	322.8 $ light^{-1}, 9 light ha^{-1}, 5 years for 1 light	581.0
**Sum**			**1,463**
**Electricity**	Pesticide spray	0.1 $ per kilowatt-hour	**40.7**
	Irrigation		26.1
**Sum**			**67**
**Materials & depreciation costs**	Apple paper bag		544.7
	The bee reed pipe		24.2
	Gasoline for mower		84.7
	Mower depreciation costs		69.6
	Spray depreciation costs		55.5
	Water pump depreciation costs		34.8

**Sum**			**814**
**Others**	Orchard lease fee	807 $ $year^{-1}$	**807**
**Total**			**12,114**

Details of apple price of different quality level

Quality level*	Conventional ($ kg^{-1})	Organic ($ kg^{-1})
1	0.97	4.84
2	0.81	3.87
3	0.65	2.58
4	0.48	0.48
5	0.32	0.32
6	0.10	0.10

*There are four levels for apple, we sold apple according to quality level.

TableS4 Detailed list of cash forest inputs and outputs for Biodiversity Management of Organic Farming (BMOF).

Details of Cash Forest Inputs			
**Content**	Details	Price Schedules	Amount ($ ha^{-1})
**Materials**	Sapling cost	1.59 $ $plant^{-1}$, 900 plant ha^{-1}	1,431.0
	Requirements for irrigation to government	217.89 $ ha^{-1}	219.9
**Sum**			**1,651**
**Labor**	Tree planting	100 labor ha^{-1}	645.6
	Irrigation	9 labor ha^{-1}	87.2
	Harvest	200 labor ha^{-1}	1,291.2
**Sum**			**2,024**
**Total**			**3,675**

Details of Cash Forest outputs*		
Content	Price Schedules	Amount($ ha^{-1})
Woods	12.91 $ $plant^{-1}$, 900 plant ha^{-1}	**11,621**

*Cash forest needs 7-8 years to grown, so we only got the harvest one time in 2014.

TableS5. Detailed lists of cattle benefits from Hongyi Organic Farm during the experimental run (2007-2014).

Years	Cattle capita	Cattle manure production(tons)	Inputs($)										Outputs($)	
			Cattle house*	Fodder store**	Residues proceeding machine***	Residues silage pool****	Fodder mixing machine*****	Weighbridge******	Young cattle buying costs	Breeding labor [∮]	Residues proceeding labor [§]	Fodder	Beef	Manure
2007	50	263	968	339	303	796	569	520	29,052	678	807	5,897	53,262	1,271
2008	70	368	968	339	303	796	569	520	40,673	678	1,130	8,255	74,705	1,779
2009	100	525	968	339	303	796	569	520	72,630	1,356	2,018	11,793	159,786	2,542
2010	120	630	968	339	303	796	569	520	87,156	1,356	2,421	14,152	213,048	3,050
2011	140	735	968	339	303	796	569	520	152,523	1,356	2,825	16,510	248,556	3,559
2012	150	788	968	339	303	796	569	520	163,418	1,356	3,632	17,690	319,572	3,813
2013	180	945	968	339	303	796	569	520	261,468	2,034	4,358	21,227	415,444	4,576
2014	200	1050	968	339	303	796	569	520	290,520	2,034	4,842	23,586	461,604	5,084

* Cow house cost was depreciation cost for 20 years, total inputs of house was19,368 $.

** Fodder store was depreciation cost for 20 years, total inputs was 6,779 $.

*** These inputs were depreciation cost for two residues proceeding machine. Total inputs was 3,034 $ for 10 years.

**** Silage pool cost was depreciation cost for 20 years, total inputs was 15,922 $.

***** Fodder mixing machine was depreciation cost for 10 years, total inputs was 5,689 $.

******Weighbridge was depreciation cost for 10 years, total inputs was 5,195 $.

[∮]Cattle breeding need average 7 month.

[§]Residues proceeding needs 2.5 labor per head

TableS6. Detailed list of chicken and goose breeding inputs and outputs in Hongyi Organic Farm during the experimental run (2007-2014).

Years	chicken capita	goose capita	Inputs($)						Outputs($)	
			houses cost*	young chicken	young goose	medicine	labor	fodder	chicken	goose
2007	1,000	1,000	194	484	1,291	161	1,162	6,391	3,669	8,312
2008	2,000	2,000	194	968	2,582	323	1,162	8,522	7,522	16,947
2009	1,000	1,000	194	484	1,291	161	1,162	6,391	202**	9,038
2010	1,000	500	194	646	807	121	2,324	4,261	4,083	4,600
2011	1,000	1,000	194	646	1,614	161	2,324	6,391	4,567	9,958
2012	1,000	1,000	194	646	1,614	161	2,324	6,391	5,456	10,088
2013	1,000	1,000	194	646	1,937	161	3,486	6,391	5,508	10,588
2014	1,000	1,000	194	646	1,937	161	3,486	6,391	5,578	10,677

*Houses cost is depreciation cost for 10 years, total inputs of house is 1,937 $.

**This year chicken dead due to diseases, only 150 chickens alive.

TableS7. Detailed price list used in different years' calculation.

Years	Labor*		Young cattle	beef	Young chicken	live chicken	Young goose	live goose
	$ day^{-1}	$ month^{-1}	$ kg^{-1}	$ kg^{-1}	$ head^{-1}	$ kg^{-1}	$ kg^{-1}	$ kg^{-1}
2007	6.46	48.42	2.58	1.94	0.48	2.72	2.58	1.66
2008	6.46	48.42	2.58	1.94	0.48	2.79	2.58	1.70
2009	8.07	96.84	3.23	2.91	0.48	2.69	2.58	1.81
2010	8.07	96.84	3.23	3.23	0.48	3.03	3.23	1.84
2011	8.07	96.84	4.84	3.23	0.65	3.38	3.23	1.99
2012	9.68	145.26	4.84	3.87	0.65	4.04	3.23	2.02
2013	9.68	145.26	6.46	4.20	0.65	4.08	3.87	2.12
2014	9.68	145.26	6.46	4.20	0.65	4.13	3.87	2.14

* Two style of payment for labor force and the price varied in different years.

TableS8. Detailed list of crop yields and apple yields in Hongyi Organic Farm during the experimental run (2007-2014).

Years	Crop yields of BMOF(kg)*		Crop yields of CF(kg) *		Apple yields in different quality levels (kg)**						
	Wheat	Maize	Wheat	Maize	1	2	3	4	5	6	Total
2007	35,260.3	41,002.5	53,298.8	55,235.2	461.3	922.5	1,845.0	3,228.8	2,306.3	461.3	9,225
2008	15,674.8	27,388.7	52,673.6	53,133.4	474.8	949.5	1,899.0	3,323.3	2,373.8	474.8	9,495
2009	31,349.7	28,349.2	54,081.7	56,378.3	510.8	1,021.5	2,043.0	3,575.3	2,553.8	510.8	10,215
2010	46,690.3	54,817.4	55,233.5	62,190.1	492.8	985.5	1,971.0	3,449.3	2,463.8	492.78	9,855
2011	48,074.0	47,653.8	52,826.0	55,571.2	495.0	990.0	1,980.0	3,465.0	2,475.0	495.0	9,900
2012	50,162.1	53,204.6	53,091.9	54,901.8	402.8	805.5	1,611.0	2,819.3	2,013.8	402.8	8,055
2013	40,763.9	35,417.7	57,857.5	51,622.3	427.5	855.0	1,710.0	2,992.5	2,137.5	427.5	8,550
2014	57,587.0	68,269.1	64,027.7	92,787.6	452.3	904.5	1,809.0	3,165.8	2,261.3	452.3	9,045

*****Field land for BMOF crop was 6.7 ha, while land for CF crop was 8.7 ha

**Apple orchard land was 0.3 ha.

TableS9. Labor inputs in the Hongyi Organic Farm during the eight-year run.

Years	Crops ($)	Orchard($)	Cattle($)	Chicken + goose($)	Cash forest($)	Total($)
2007	5,627	1,337	1485	1,162	282	9,893
2008	5,627	1,337	1808	1,162	0	9,934
2009	7,029	1,670	3374	1,162	0	13,235
2010	7,029	1,670	3777	2,324	0	14,800
2011	7,029	1,670	4181	2,324	0	15,204
2012	8,431	2,004	4988	2,324	0	17,747
2013	8,431	2,004	6392	3,486	0	20,313
2014	8,431	2,004	6876	3,486	774	21,571
